# Molecular characterisation of methicillin-resistant Staphylococcus aureus (MRSA) isolated at a large referral hospital in Zambia

**DOI:** 10.11604/pamj.2017.26.108.10982

**Published:** 2017-02-28

**Authors:** Mulemba Tillika Samutela, Annie Kalonda, James Mwansa, Chileshe Lukwesa-Musyani, John Mwaba, Enoch Mulowa Mumbula, Darlington Mwenya, Edgar Simulundu, Geoffrey Kwenda

**Affiliations:** 1 Department of Biomedical Sciences, School of Medicine, University of Zambia,P.O. Box 50110, Lusaka, Zambia; 2 Department of Pathology and Microbiology, University Teaching Hospital, P/Bag RW X1 Lusaka, Zambia; 3 Department of Pathology and Microbiology, School of Medicine, University of Zambia, P.O. Box 50110, Lusaka, Zambia; 4 Department of Disease Control, School of Veterinary Medicine, University of Zambia, P.O. Box 32379, Lusaka, Zambia

**Keywords:** MRSA, pvl, SCCmec typing, spatyping, Zambia

## Abstract

**Introduction:**

Methicillin-resistant *Staphylococcus aureus* (MRSA) is globally recognized as an important public health problem. Whereas comprehensive molecular typing data of MRSA strains is available, particularly in Europe, North America and Australia, similar information is very limited in sub-Saharan Africa including Zambia.

**Methods:**

In this study, thirty two clinical isolates of *Staphylococcus aureus*, collected at a large referral hospital in Lusaka, Zambia between June 2009 and December 2012 were analysed by Staphylococcal cassette chromosome *mec (SCCmec), Staphylococcus* protein A gene typing *(spa)* and detection of the Panton-Valentine Leukocidin genes *(pvl)*.

**Results:**

Three SCC*mec* types were identified namely SCC*mec* type IV (65.6%), SCCmec type III (21.9%), SCC*mec* type I (3.1%). Nine point four percent (9.4%) of the isolates were untypable. Five *spa* types, which included a novel type, were detected and the most prevalent spa type was t064 (40.6%). Other *spa* types included spa types t2104 (31.3%), t355 (3.1%) and t1257 (21.9%). The *pvl* genes were detected in 3 out of 32 isolates.

**Conclusion:**

These molecular typing data indicated that the MRSA strains collected in Lusaka were diverse. Although the source of these MRSA was not established, these results stress the need for assessing infection prevention and control procedures at this health-care facility in order to curtail possible nosocomial infections. Furthermore, country-wide surveillance of MRSA in both the community and health-care facilities is recommended for infection prevention and control. To our knowledge, this represents the first study to characterise MRSA using molecular tools in Zambia.

## Introduction

Methicillin-resistant *Staphylococcus aureus* (MRSA) has been recognised as one of the major causes of nosocomial or hospital acquired infections worldwide [1]. Methicillin resistance is due to the acquisition of genes encoding a unique penicillin-binding protein namely PBP2a. PBP2a has decreased affinity for β-lactams and catalyses effective cell wall synthesis even in the presence of penicillins, cephalosporins and carbapenems [2–4]. PBP2a is encoded by the *mec* A gene which is carried on a mobile element known as the staphylococcal cassette chromosome *mec* (SCC*mec*) [3–6]. Several SCC*mec* subtypes and their variants have been characterised [4–6]. Currently there are twelve subtypes (i.e. SCC*mec* type I-XII) that have been identified [7, 8]. Since the discovery of MRSA in the United Kingdom (UK) in the early 1960s [9], its prevalence has steadily increased in the world with geographical variations [4, 10, 11]. The burden of MRSA infections has further been heightened by the emergence of community-acquired MRSA (CA-MRSA) and livestock-acquired MRSA (LA-MRSA) [12, 13]. While the clonal relatedness of MRSA isolates from developed countries has been extensively analysed, there is sparse information from developing countries [14]. *Staphylococcus aureus*, including MRSA have several virulence factors that contribute to the pathogenicity of the organism. One such virulence factor is the Panton-Valentine leukocidin (PVL) toxin encoded by two genes, *lukF-PV* and *lukS-PV*, carried on lysogenic bacteriophages. PVL is associated with CA-MRSA and is linked to skin and soft tissue infections (SSTIs). However, *pvl* genes have also been detected in Health-care associated MRSA (HA-MRSA) isolates [15].

Generally, the prevalence rates of MRSA from most African countries have been shown to range from 25% to 50% [16]. Studies conducted in South Africa, Nigeria, Kenya, and Cameroon found the prevalence ranging from 21 to 33.3% [17, 18]. However, other studies conducted in Tunisia, Malta, and Algeria found the prevalence was below 10% [18]. A more recent study from Kenya found the MRSA prevalence rate of 84.1% among SSTIs which was higher than previous findings in the region [19]. These observations seem to suggest a trend of increasing MRSA cases over the years and hence the need for continued monitoring and control of MRSA infections in Africa. In Zambia, a similar pattern of increasing number of MRSA cases over the years has been observed. For example, at the University Teaching Hospital (UTH), a tertiary referral and teaching hospital in Lusaka, the prevalence of MRSA among *Staphylococcus aureus*isolates was found to be 23% and 30% in 2003 and 2010, respectively [20, 21]. Moreover, the most recent studies conducted at UTH in 2012 and 2014 estimated the prevalence of MRSA at 37% and 43% respectively [22, 23]. However, no systematic studies have been carried out to understand the extent of the problem of MRSA in Zambia. In a previous study, we determined that the MRSA strains isolated at UTH between 2009 and 2012 were highly multi-drug resistant [23]. However, the molecular characteristics of the MRSA strains were unknown. The aim of this study was to determine the molecular characteristics of MRSA isolated at UTH using SCC*mec* typing, spa typing and detection of *pvl* genes.

## Methods

**Study design and setting:** The study was a laboratory-based cross-sectional study conducted at UTH in the Bacteriology Laboratory in the Department of Pathology and Microbiology.

**Bacterial isolates:** A total of 32 MRSA isolates collected from June, 2009 to December, 2012 at UTH, the largest referral hospital and the centre for all microbiology diagnostic work in Zambia were included in the study. These isolates were obtained from pus and blood samples. The clinical isolates were first plated onto Columbia blood agar plates (Mast Group Ltd, Merseyside, UK) and incubated at 37°C for 24 hours. *Staphylococcus aureus* isolates were identified by standard microbiological methods including colony morphology, Gram stain, catalase reaction, coagulase activity and DNAse test as previously described [23]. Resistance to methicillin was detected using oxacillin and cefoxitin discs using the Kirby- Bauer disc diffusion method as previously described [23].

**DNA extraction:** The NucliSENS easyMAG nucleic acid extraction protocol (bioMérieux Inc, Durham, NC, USA), was used to extract the genomic DNA. Briefly, the bacterial isolates were cultured overnight on blood agar at 37°C. Using a loop, 5 bacterial colonies of a pure culture were emulsified in 700µl of NucliSENS easyMAG lysis buffer in an eppendorf tube and left to stand for 30 minutes to one hour at room temperature for maximum off-board lysis. Then 400µl of the bacterial suspension was transferred to the easyMAG disposable sample strip wells and 100µl of undiluted silica were added to the sample-lysis buffer mix. The sample strips were then loaded onto the easyMAG machine 3.2 v3 system (bioMérieux Inc, Durham, NC, USA) and the NucliSENS easyMag off-board lysis procedure was followed according to the manufacturer's instruction to extract the DNA. Staphylococcus cassette chromosome mec typing To determine the SCC*mec* structural variants of each MRSA isolate, a previously described protocol for a SCC*mec* multiplex PCR [24, 25] was used. Plasmid DNA containing the SCC*mec* type I-IV was used as controls.

**Spa typing:** *Spa* typing was done following a previously described protocol [26]. *Staphylococcus aureus* ATCC 25923 was used as the control strain.

**DNA sequencing:** Sequencing of the protein A gene (*spa*) was performed at the Inqaba Biotechnical Industries Sequencing Facility (Pretoria, South Africa) using BigDye terminator method with an ABI PRISM 3730XL DNA analyser (Applied Biosystems, Foster City, CA, USA). The DNA sequence reads were edited using the Ridom TraceEdit Software (Ridom Bioinformatics GmbH, Würzburg, Germany).

**Spa type determination and phylogenetic analysis:** The *spa* types were determined using the software package Bionumerics *Spa* typing plugin version 7.1 (Bionumerics, Belgium) after in putting the chromatograph sequence files of the isolates. The phylogenetic and minimum spanning trees were also generated using the same software.

**PVL genes detection:** To detect the *pvl genes*, a previously described protocol was followed [27]. A previously known *pvl gene* positive *Staphylococcus aureus* isolate was used as a control strain.

## Results

**Staphylococcus cassette chromosome *mec* types:** The *mec* A gene was detected in all the 32 isolates. Three SCC*mec* types were identified namely SCC*mec* type IV (65.6%), SCC*mec* type III (21.9%) and SCC*mec*type I (3.1%). Nine point four percent of the isolates were untypable.

**Figure 1:**
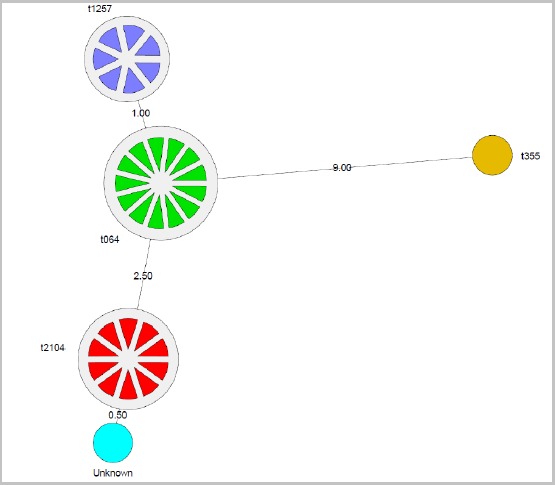
Minimum spanning tree showing the relationship of the isolates in relation to the most frequent *spa* type determined (t064): the different colours represent the *spa* types namely green for t064, red for t2104, blue for t1257, orange for t355 and sky blue for the unkown *spa* type

**Staphylococcal protein A (*spa*) types :** The MRSA strains were found to be of 5 spa types namely t064 (40.6%), t2104 (31.3%), t355 (3.1%), t1257 (21.9%) and unknown *spa* type (3.1%) as shown in **Table 1**. Phylogenetic analysis of the MRSA strains based on the *spa* types showed that isolate MS09 with *spa* type t355 was most distantly related when compared with the rest of the strains characterised. Also all the other isolates separated in two major groups. The first group included isolates with *spa* types t1257 and t064 while the other group included isolates with *spa* type t2104 and the novel *spa* type. A minimum spanning tree confirmed these relationships (**Figure 1**). **PVL genes:** the PVL genes were detected in 9.4% of the isolates (3/32).

**Table 1:** Distribution of Spa types among the MRSA isolates (n=32)

Proportion of isolates % (n)	*Spa* type	Repeat Succession
40.6 (13)	t064	11-19-12-05-17-34-24-34-22-25
31.3 (10)	t2104	11-19-12-34-24-34-22-25
3.1 (1)	t355	07-56-12-17-16-16-33-31-57-12
21.9 (7)	t1257	11-19-34-05-17-34-24-34-22-25
3.1 (1)	Unknown	11-19-12-34-24-34-22-25-25

## Discussion

The present study reports the molecular characteristics of MRSA isolated at the largest referral and teaching hospital in Zambia from 2009 to 2012. Three SCC*mec* types were found, the most predominant being type IV which is usually associated with CA-MRSA. However, it has been increasingly found in HA-MRSA [28, 29]. Moreover, the HA-MRSA strains with SCC*mec* type IV are multidrug resistant while CA-MRSA strains are generally more susceptible [28]. The MRSA isolates studied here were multi-drug resistant and the presence of the SCC*mec* types III and I confirmed this multidrug resistance as previously reported [23]. The findings of our study are similar to those of a study from South Africa in which the second predominant SCC*mec* type was type III [30]. SCCmec types I and III are normally harboured by HA-MRSA [31–33]. HA-MRSA strains containing SCC*mec* type III tend to be multi-drug resistant since this SCC*mec* type is known to harbour plasmids and transposons that encode resistance to tetracycline, cadmium, erythromycin and spectinomycin [6, 34–36]. The 9.4% of isolates detected in this study that were SCC*mec* non-typable is comparable to the 8% from the South African study [30] but slightly higher when compared to those from a study in Belgium (4%) [37].

In determining the *spa* types, 5 *spa* types were found and the most prevalent was t064 which was found in 40.6% of the isolates. *Spa* type t064 has been identified in the USA where it was designated ST-8 ORSA I and was also associated with persons living with AIDS [38, 39]. A study in Nigeria also found *spa* type t064 as the most predominant spa type in HIV positive *Staphylococcus aureus* carriers [40]. However, we could not associate our isolates belonging to this *spa* type with any condition or infection due to lack of patients' data. *Spa*types t2104 (31.3%) and t1257 (21.9%) were the second and third most common *spa* types identified in our study, respectively. We could not find other studies documenting *spa* type t2104 among MRSA isolates despite the relatively high frequency of t2104 in our study. However, according to data available on the *spa* server, this particular spa type has been reported in association with MRSA in the United States, Sweden and Japan [41]. Notably, *spa* type t2104 was reported among Methicillin susceptible *Staphylococcus aureus* isolates in a study to determine the prevalence and molecular epidemiology of *Staphylococcus aureus* among rural Iowans, including individuals with livestock contact [42]. *Spa* type t1257 has been reported in South Africa and it accounted for about 9.7% of the isolates [43]. Spa type t1257 has been associated with HA-MRSA strains. The only singleton *spa* type found in this present study was *spa* type t355. Although this *spa* type is rarely reported, it was the most prevalent *spa* type identified among MRSA isolates in a study from Ghana [44]. It has also been documented in Nigeria and Uganda [45, 46].

Only one novel *spa* sequence was determined in our study. It has not been reported previously from any other country and its name could not be determined by the *spa* sever. Although this novel *spa* type seems to be closely related to *spa* type t2104, its repeat sequence contains two repeats r25 end and is shorter than the repeat sequence for t2104. This finding possibly denotes mutations in the S. aureus genome. *Spa* typing also allows for the grouping of isolates into groups called *spa*-clonal complex (*spa*-CC) [47]. When examining the spa types belonging to a specific *spa*-CC, there is usually a repeat or several repeats that all the spa types have in common [47]. It is generally accepted that MRSA strains are related, i.e. belong to the same *spa*-CC, if the spa type repeat motif is related [47]. From literature, we could deduce that most of our isolates belong to the *spa*-CC 064 [38–40, 48]. This is because alignment of the repeat patterns of *spa* types t064, t1257 and t2104 showed the presence of both motifs 11-19-, (start); followed by motif 05-17-34-; followed by motif 24- 34-22; and finally repeat r25 (end). However, *spa*type t2104 has relatively fewer repeats since it lacks the motifs 05-17-. In addition the repeat sequence of the novel spa type is also very similar to that of *spa* type t2104. It is worth noting that *spa*-CC 064 is associated with HA-MRSA [38, 39, 48].

The prevalence of the *pvl* genes in the present study among MRSA isolates was very low. Data from other studies conducted in Africa have shown that the proportion of PVL-positive MRSA carriage and/or infections ranges from 0.3 to 100% in humans [49]. Studies from Algeria and Tunisia reported higher PVL prevalence while investigations from South Africa reported the lowest prevalence [49]. PVL-positive MRSA is more frequently reported with SSTIs, and community-associated clones. The presence of PVL-positive MRSA could present a significant challenge in disease management and infection control in resource-limited countries such as Zambia. Therefore there is need to conduct more studies on the carriage of *pvl* genes in *Staphylococcus aureus* in Zambia. Although the lack of clinical data limited our conclusions on the possible origin/source of MRSA infections as well as with respect to disease severity or treatment outcome, our results intimate on the need for evaluating infection prevention and control procedures at UTH and possibly other health facilities in the country Therefore, further work is warranted to better understand the epidemiology of *Staphylococcus aureus*infections in humans in Zambia for institution of effective prevention and control strategies and patient care.

## Conclusion

In conclusion, these data appear to indicate that most of the strains studied may have been hospital acquired and hence the need for close examination of infection prevention and control procedures at UTH. Also, the present study underscores the need for country-wide monitoring of MRSA in both community and hospital settings for infection control.

### What is known about this topic

- Methicillin resistance is due to the acquisition of genes encoding a unique penicillin-binding protein (PBP2a) namely *mecA* and most recently *mec*C;
- MRSA is one of the major causes of nosocomial infections and has become prevalent in the community.

### What this study adds

- The molecular characteristics of MRSA isolates from Zambia have been determined;
- One novel spa type has been discovered.
